# Comparison of Graphitic Carbon Nitrides Synthetized from Melamine and Melamine-Cyanurate Complex: Characterization and Photocatalytic Decomposition of Ofloxacin and Ampicillin

**DOI:** 10.3390/ma14081967

**Published:** 2021-04-14

**Authors:** Petr Praus, Aneta Smýkalová, Kryštof Foniok

**Affiliations:** 1 Department of Chemistry VŠB-Technical, University of Ostrava, 17. listopadu 15, 708 00 Ostrava-Poruba, Czech Republic; aneta.smykalova@vsb.cz (A.S.); krystof.foniok@vsb.cz (K.F.); 2 Institute of Environmental Technology, VŠB-Technical, University of Ostrava, 17. listopadu 15, 708 00 Ostrava-Poruba, Czech Republic

**Keywords:** graphitic carbon nitride, melamine, melamine-cyanurate complex, photocatalysis, ofloxacin, ampicillin

## Abstract

Graphitic carbon nitride (g-C_3_N_4_, hereafter abbreviated as CN) was prepared by the heating of melamine (CN-M) and melamine-cyanurate complex (CN-MCA), respectively, in air at 550 °C for 4 h. The specific surface area (SSA) of CN-M and CN-MCA was 12 m^2^ g^−1^ and 225 m^2^g^−1^ and the content of oxygen was 0.62 wt.% and 1.88 wt.%, respectively. The band gap energy (E_g_) of CN-M was 2.64 eV and E_g_ of CN-MCA was 2.73 eV. The photocatalytic activity of the CN materials was tested by means of the decomposition of antibiotics ofloxacin and ampicillin under LED irradiation of 420 nm. The activity of CN-MCA was higher due to its high SSA, which was determined based on the physisorption of nitrogen. Ofloxacin was decomposed more efficiently than ampicillin in the presence of both photocatalysts.

## 1. Introduction

Graphitic carbon nitride (CN) is a metal-free semiconducting material that has been intensively studied during the last decade. Its mechanical, chemical, and thermal stability and capability to absorb visible irradiation (band gap energy of about 2.7 eV) predetermine this material for many medical [1,2], industrial [3,4], and environmental applications [5,6,7,8,9]. Graphitic carbon nitride can be synthetized from simple nitrogen-rich organic compounds such as melamine, cyanamide, dicyanamide, urea, thiourea, etc. CN based materials can be used as photocatalysts [10,11], biosensors [12] and chemical sensors [13] and as materials for biomedical applications [14], energy and environmental applications [4], etc.

Herein, we report on a comparison of CN synthetized from common melamine and from the complex of melamine and cyanuric acid called melamine-cyanurate (MCA). Melamine (M) and cyanuric acid (CA) form an insoluble MCA complex [15] as follows:

M + CA ⇆ MCA(1)

The solubility of MCA in water is S = 31 μmol L^−1^ [16] and the solubility product is K_s_ = S^2^ = 9.6 × 10^−10^. The MCA complex is capable of self-assembly, forming a monolayer network stable up to 350 [17] or 450 °C [18]. In this complex, M and CA are connected by means of the hydrogen bonding of N-H…O and N-H…N [19]. Three-dimensional (3D) structures of CN are formed from MCA: spheres [17,19,20,21], tubes [22,23,24], wires [25], flowers [26,27], seaweed [28], holey structures [29,30], etc.

The aim of this work was to synthetize CN from MCA and test it for the photocatalytic decomposition of antibiotics ofloxacin and ampicillin. CN commonly synthetized from melamine was used for comparison. The differences between both CN materials were discussed. Ofloxacin (±-9-Fluoro-2,3-dihydro-3-methyl-10-(4-methyl-1-piperazinyl)-7-oxo-7H-pyrido[1,2,3-de]-1,4-benzoxazine-6-carboxylicacid) (Supplementary Materials, Figure S1) is a fluoroquinolone antibiotic with superior antimicrobial properties and acts through the inhibition of bacterial gyrase, an enzyme involved in DNA replication, recombination, and repair. However, they have been associated with disabling and irreversible serious adverse reactions (tendinitis and tendon rupture, peripheral neuropathy, central nervous system effects, etc.) that have occurred [31,32]. Ampicillin ((2S,5R,6R)-6-[[(2R)-2-amino-2-phenylacetyl]amino]-3,3-dimethyl-7-oxo-4-thia-1-azabicyclo[3.2.0]heptane-2-carboxylic acid) (Supplementary Materials, Figure S2) is a broad-spectrum beta-lactam penicillin antibiotic with bactericidal activity. It has been used since 1961 to treat many different types of infections caused by bacteria such as ear infections, bladder infections, pneumonia, gonorrhea, and *E. coli* or salmonella infection, etc. [33,34]. Ampicillin is less toxic than other antibiotics and some side effects are observed when persons are sensitive to penicillins. In general, such kinds of antibiotics have been largely consumed and get into wastewaters where they could be a serious problem for the environment [35,36,37]. New water treatment technologies for their effective removal need to be developed.

The novel finding of this work is that CN-MCA formed a very porous material with high specific surface area and there was no need for its further exfoliation. In addition, CN-MCA was not found to create various 3D objects as described in the literature. The application of CN-MCA for the decomposition of the antibiotics is also novel.

## 2. Materials and Methods

### 2.1. Chemicals

All chemicals used were of analytical-reagent grade. Melamine, cyanuric acid, ofloxacin, and ampicillin were purchased from Sigma-Aldrich (Darmstadt, Germany). Deionized water with the conductivity of 0.1 μS cm^−1^ was used for the preparation of all solutions and experiments.

### 2.2. Synthesis of Melamine-Cyanurate (MCA)

The supramolecular complex of melamine and cyanuric acid was prepared by dissolving 0.25 g of melamine and 0.25 g of cyanuric acid in water in separate beakers and then mixing them together to form MCA. Redundant water was removed by drying at 105 °C until constant weight.

### 2.3. Synthesis of melamine (CN-M) and Melamine-Cyanurate Complex (CN-MCA)

CN-M was prepared by heating melamine in air in a ceramic crucible with a lid (diameter 5 cm, 30 mL), starting from room temperature with the heating rate of 3 °C min^−1^ up to 550 °C. Then, the temperature was kept at 550 °C for 4 h. The final product was cooled down to room temperature out of the muffle furnace. CN-MCA was prepared by the heating of MCA by the same procedure and under the same conditions as CN-M above-mentioned.

### 2.4. Elemental Analysis

The contents of C, N, and H in the CN materials were determined by means of a Flash 2000 Elemental analyzer (ThermoFisher Scientific, Waltham, MA, USA). The content of oxygen was calculated as a difference to 100%.

### 2.5. UV–Vis Spectrometry

UV–Vis diffuse reflectance (DR) spectra were obtained using a Shimadzu UV-2600 spectrophotometer (IRS-2600Plus, Shimadzu, Kjóto, Japan). Measured reflectance values were transformed to Schuster–Kubelka–Munk’s ones as follows

(2) $$F\left(R_{\infty }\right)=\frac{{\left(1-R_{\infty }\right)}^{2}}{2R_{\infty }}$$

where *R_∞_* is the diffuse reflectance of a semi-infinite layer.

### 2.6. Specific Surface Area Measurement

Physisorption of nitrogen was measured using a Sorptomatic 1990 instrument (Thermo Fischer Scientific Inc., Electron Corporation, Waltham, MA, USA) after sample degassing at room temperature for 48 h under less than 1 Pa vacuum. The adsorption-desorption isotherms of nitrogen were measured at 77 K. SSA was evaluated according to the classic Brunauer–Emmett–Teller (BET) theory for the p/p^0^ = 0.05–0.25. Pore-size distribution was calculated from the adsorption branch of the nitrogen adsorption-desorption isotherm using the Barrett, Joyner, and Halenda (BJH) method, the empirical Broekhoff–De Boer standard isotherm, and assuming the cylindrical pore geometry.

### 2.7. X-ray Diffraction Analysis

The X-ray diffraction (XRD) patterns were recorded by means of a Rigaku SmartLab diffractometer (Rigaku, Tokyo, Japan) with a detector D/teX Ultra 250. A source of X-ray irradiation was a Co tube (CoKα, *λ*_1_ = 0.178892 nm, *λ*_2_ = 0.179278 nm) operated at 40 kV and 40 mA. The X-ray diffractograms were recorded between 5° and 90° of 2θ with the step size of 0.01° and speed of 0.5 deg min^−1^. The crystallite size *L* was calculated using Scherrer’s equation for broadening *B*(2θ) (in radians) at a half maximum intensity (FWHM) of a diffraction band as

(3) $$B\left(2\Theta \right)=\frac{K\lambda }{Lcos\Theta }$$

where *λ* is the wavelength of X-rays; θ is Bragg´s angle; and *K* is the constant equal to 0.94 for cube or 0.89 for spherical crystallites. In this work, *K* = 0.9.

### 2.8. Fourier Transform Infrared Spectroscopy

The Fourier transform infrared (FTIR) spectra were recorded using a Nicolet iS50 device (Thermo Scientific, Waltham, MA, USA) by the KBr technique. A small amount of sample was mixed and homogenized with KBr (approximately 200 mg) and pressed to obtain a transparent tablet. Each spectrum consisted of at least 64 scans.

### 2.9. Scanning Electron Microscopy

Scanning electron microscopy (SEM) was performed by a Tescan Vega microscope (Tescan Orsay Holding, Inc., Brno, Czech Republic). The SEM micrographs were obtained using secondary electrons and backscattered electron mode with an acceleration voltage of 30 keV. The CN samples before imaging were gold sputtered in order to ensure adequate electron conductivity.

### 2.10. Photocatalytic Decompositions

The photocatalytic activity of the CN materials was investigated using ofloxacin and ampicillin in the concentration of 20 mg L^−1^ (both). A total of 45 mg of each CN material was added into 150 mL of this solution and stirred for 60 min to reach adsorption-desorption equilibria. Then, the suspension was irradiated with a LED source (420 nm) with the intensity of 7.1 mW cm^−2^. The samples were analyzed by a high-performance liquid chromatograph (HPLC) Waters 2996 (Waters Corporation Milford, MA, USA) with a PDA detector. A column Synergi 4 µm Polar-RP 80 Å (100 × 3 mm) was used for the separation. The mobile phase consisted of acetonitrile and 0.04 mol L^−1^ chloroacetic acid (25:75, *v*/*v*) with pH adjusted to 3 using NH_4_OH.

### 2.11. Electrochemical Measurements

Electrochemical measurements were performed using a Metrohm Autolab PGSTAT302 (Herisau, Switzerland) potentiostat. A glassy carbon electrode (GC), Ag/AgCl (3 mol L^−1^ KCl) electrode and a Pt sheet served as working, reference, and counter electrodes, respectively. All electrodes were purchased from Metrohm (Herisau, Switzerland). A thin layer of CN samples was prepared on the GCE surface by the following procedure.

Powdered samples, each in the amount of 10 mg, were added to 5 mL of deionized water, and then the mixtures were subjected to 30-min sonication in an ultrasonic bath. Then, 30 μL of the dispersion was dropped on the GC surface and dried for 3 h at 85 °C. The samples were measured in 0.1 mol L^−1^ KCl aqueous solution, which was purged with nitrogen for 30 min before the experiment. Mott–Schottky measurements were performed twice with an AC signal having an amplitude of 10 mV and a frequency of 300 Hz.

### 2.12. Zeta Potential Measurement

The zeta potentials of the CN samples were measured using a Malvern Zetasizer Ultra (Malvern Instruments Ltd., Worcestershire, UK). Before each analysis, the solid sample (5 mg) was dispersed in 50 mL of deionized water by ultrasonication for 5 min. The dispersed sample was placed in a sample container, which was then attached to a MPT-3 Multi-purpose titrator and titrated. A folded capillary cell DTS1070 was used for the zeta potential measurement, which was performed by a ZS XPLORER program using an automated titration system (titrator MPT-3, pH electrode type MV 114-S.C. SEN 0106, Malvern Instrument Ltd. (Worcestershire, UK); vacuum degasser, P/N, 8700-3480v3, Systec).

### 2.13. Statistical Calculations

Statistical calculations including linear regression were performed at the α = 0.05 significance level using the software package OriginPro 2018b, ver. B9.5.5.409 (OriginLab Corporation, Northampton, MA, USA).

## 3. Results

The CN materials were prepared by heating melamine and the melamine-cyanurate complex. Their physico-chemical properties were studied by means of common characterization methods. Their photocatalytic activity was investigated by the photocatalytic decomposition of ofloxacin and ampicillin.

### 3.1. Elemental Analysis

Using the different precursors of CN such as melamine and MCA, the composition of final graphitic carbon nitrides was determined for comparison. The elemental composition of the CN materials was performed as summarized in Table 1. Elemental analysis was used for the determination of C, H, and N. The precipitated MCA without further heating was analyzed for comparison. The remarkable decrease of the oxygen content between MCA and CN-MCA is given by dehydroxylation during the MCA heating. Oxygen in CN-M exists due to oxidation during the melamine heating in air [38]. The contents of nitrogen and hydrogen in CN-MCA were only a little higher, likely to be due to their dihydroxylation, which is also the reason why the C/N values of both materials were only a little different.

### 3.2. UV–Vis DR Spectroscopy

**Figure 1:**
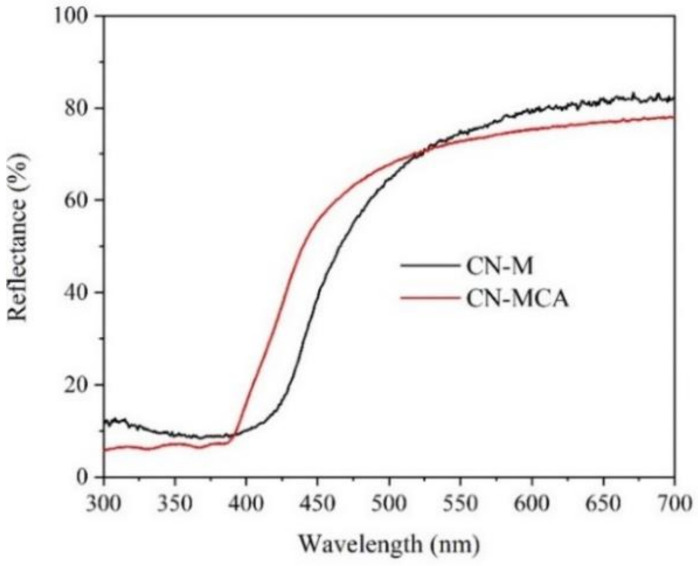
UV–Vis DR spectra of graphitic carbon nitride (CN) materials.

The UV–Vis spectra of CN-M and CN-MCA are demonstrated in Figure 1. The blue shift of CN-MCA toward CN-M indicates their different properties. The blue shift is documented by the band gap energies, which were evaluated by means of the commonly employed Tauc’s plot defined as:

(4) $$\varepsilon h\nu =C{\left(h\nu -E_{g}\right)}^{p}$$

where *ε* is the molar extinction coefficient; *hν* is the energy of incident photons; *C* is a constant; and *p* is the power, which depends on the type of electron transition: *p* = 2 was used in this study. The band gap energies are given in Table 2.

In this table, the values of SSA are also given. The SSA of CN-MCA was even higher than that of CN-M exfoliated into nanosheets [39]. In general, the increase of *E_g_* was found to be caused by exfoliation [39,40], therefore, the CN-MCA structure exfoliated into the nanosheets could be the reason for the blue shifted light absorption. This could be caused by the dehydroxylation of MCA during its heating, as above-mentioned.

### 3.3. Specific Surface Area and Pore Size Distribution Measurement

**Figure 2:**
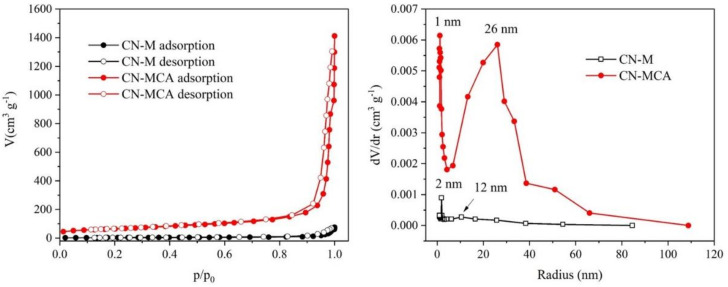
Adsorption and desorption isotherms of nitrogen at 77 K (**left**) and distribution curves (**right**) of CN-M and CN-MCA.

The physisorption of nitrogen was measured to obtain the adsorption and desorption isotherms, which were evaluated using the BET isotherm. The isotherms shown in Figure 2 clearly demonstrate the higher adsorption on CN-MCA. In both materials, the mesoporous structure was indicated by hysteresis loops (not well visible for CN-M). The cumulative mesopore volumes were 32 cm^3^g^−1^ and 1.7 cm^3^g^−1^ for CN-MCA and CN-M, respectively.

The pore size distribution in CN-M and CN-MCA is also shown in Figure 2. The distribution curves are broad, implying the presence of mesopores and macropores with the radii up to 90–100 nm. The CN-MCA material contained more pores than the CN-M one, which agrees with its high specific surface area. The most frequent pore radii of about 2 nm and 1 nm in CN-M and CN-MCA, respectively, were observed. Both distribution curves also demonstrated the presence of the second most frequent pore radii of 12 nm and 26 nm in CN-M and CN-MCA, respectively.

### 3.4. XRD Analysis

**Figure 3:**
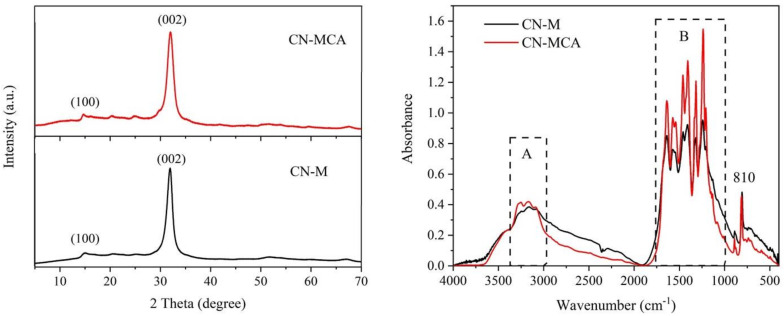
X-ray diffraction (XRD) patterns (**left**) and Fourier transform infrared (FTIR) spectra (**right**) of CN materials.

The material structures were studied by means of XRD, as demonstrated in Figure 3. Two low intensity diffraction peaks at 2Θ at around 15° and 32° corresponding to (100) and (002) diffractions were found. These were assigned to the hexagonal phase of CN (JCPDS 87-1526). The more intensive (002) diffraction corresponded to interlayer arrangement of (002) melem planes and the less intensive (001) one corresponded to in-plane ordering of connected heptazine units [41,42].

The d-spacings of the (002) planes d(002) were the same for both materials. The crystallite sizes L(002) and FWHM(002) values corresponding to the (002) diffractions were different (see Table 3) due to the partial exfoliation of CN-MCA into nanosheets, but less than was referred in the literature [43].

### 3.5. FTIR Analysis

The structure of CN-M and CN-MCA was investigated by means of FTIR spectrometry, as displayed in Figure 3. Two regions, A and B, typical of graphitic carbon nitride were observed. The bands in region A ere associated with the stretching vibrations of N–H bonds and bands in region B were associated with the stretching vibrations of C=N and C–N bonds of heterocyclic rings [44]. The narrow bands around 810 cm^−1^ were associated with the breathing mode of triazine units [45]. The spectral peaks around 3500 cm^−1^ were explained by the stretching vibrations of –OH groups. The FTIR spectra of both CN materials were similar and typical of graphitic carbon nitride. On the whole, the FTIR spectra as well as the XRD patterns identified graphitic carbon nitride in the prepared materials.

### 3.6. Scanning Electron Microscopy (SEM) Analysis

**Figure 4:**
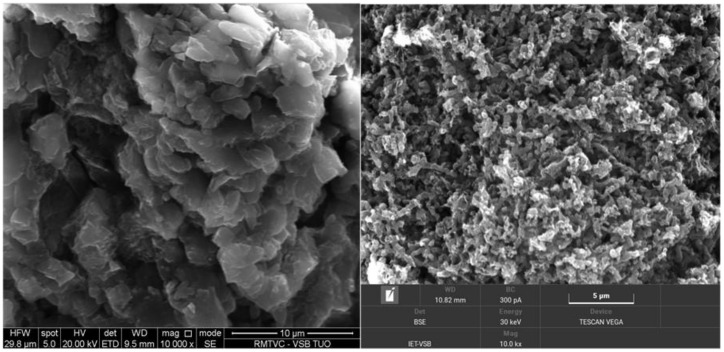
SEM micrographs of CN materials. (**left**) CN-M, (**right**) CN-MCA.

Morphology of the CN-M and CN-MCA was observed by SEM (see Figure 4). CN-MCA had a more porous structure, which agreed with its high SSA (Table 2). The reason is likely to be the dehydroxylation of MCA during its heating, which resulted in the CN-MCA exfoliation and the formation of nanosheets. The CN nanosheets were agglomerated into randomly oriented crystallites (Table 3), which were parts of particles of irregular shapes visible in the SEM micrographs. Unlike the papers mentioned in the Introduction, some 3D structures based on MCA were not observed.

### 3.7. Photocatalytic Activity

The photocatalytic activity of CN-M and CN-MCA was investigated using the antibiotics ofloxacin and ampicillin. In the dark, the suspensions of the CN materials and the antibiotics were stirred for 60 min to reach adsorption-desorption equilibrium.

The photocatalytic decomposition was supposed to be performed based on reactions with radicals formed by the complex reactions of photoinduced electrons and holes with oxygen and water, as can be found elsewhere [46,47,48]. The heterogeneous reactions of these antibiotics and radicals on the surface of CN-M and CN-MCA are possible to express by the Langmuir–Hinshelwood model and the reaction rate *r* can be defined as

(5) $$r=-\frac{dc_{A}}{dt}=k\frac{K_{A}c_{A}}{1+K_{A}c_{A}+\sum K_{i}c_{i}}\frac{K_{R}\;c_{R}}{1+K_{R}c_{R}}$$

where *k* is a kinetic parameter; *K_A_*, *K_R_*, *K_i_* and *c_A_*, *c_R_*, *c_i_* are adsorption constants and concentrations of antibiotics, radicals, and intermediates, respectively. If *c_R_* >> *c_A_* and the terms *ΣK_i_c_i_* and *K_A_c_A_* can be neglected, then Equation (5) can be simplified to its mostly used form of the first-order reaction as follows

(6) $$r=-\frac{dc_{A}}{dt}=k_{app}\frac{K_{A}c_{A}}{1+K_{A}c_{A}}=k_{obs}c_{A}$$

where *k_app_* and *k_obs_* are apparent and observed kinetic parameters, respectively, depending on irradiation intensity, mass, and nature of the solid phase (photocatalyst) and the concentration of radicals. In contrast, if *c_R_* << *c_A_* and *ΣK_i_c_i_* and *K_R_c_R_* can be neglected, then Equation (5) is simplified to the zero-order reaction as follows:

(7) $$r=-\frac{dc_{A}}{dt}=k_{app}\frac{K_{R}c_{R}}{1+K_{R}c_{R}}=k_{obs}$$

where *k_obs_* is constant, supposing that the concentration of radicals is constant, which is possible when an irradiating flux is constant.

**Figure 5:**
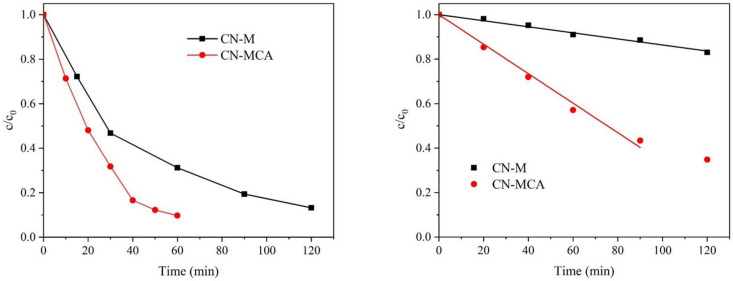
Photocatalytic decomposition of ofloxacin (**left**) and ampicillin (**right**) depending on time.

The ofloxacin and ampicillin kinetic curves demonstrated in Figure 5 indicate the first-order and zero-order reactions, respectively. The kinetic constants were evaluated and summarized in Table 4. It is remarkable that the decomposition efficiency with CN-M was always lower than that with CN-MCA, which can simply be explained by the larger SSA of CN-MCA. In addition, the decomposition of ofloxacin was more effective than that of ampicillin.

The first-order decomposition reaction of ofloxacin was also observed in the presence of CN synthetized from dicyandiamide [49] and mesoporous CN [50] under simulated sunlight. The photocatalytic decomposition of ampicillin using CN has not been described in the literature yet.

In order to consider the reaction of photoinduced holes and electrons, the Mott–Schottky method was applied to determine the conduction band potentials (E_CD_). They were measured against the Ag/AgCl reference electrode and recalculated to be against the normal hydrogen electrode (NHE) at pH = 7, as given in Table 4 [51]. The valence band potentials (E_VB_) were calculated according to the equation

E_VB_ = E_CB_ + E_g_ (8)

Comparing these results with the standard redox potentials of superoxide and hydroxyl radicals E*^o^*(OH•/H_2_O) = 2.74 V and E*^o^*(O_2_/O_2_^•−^) = −0.33 V at pH = 7 [52], one can see that the reaction of holes with water cannot be performed, unlike the reaction of electrons with oxygen, in which superoxide radicals are formed. Their formation was confirmed by electron paramagnetic resonance (EPR) in our previous work [53]. Therefore, the reactions of superoxide radicals forming hydrogen peroxide (E*^o^*(H_2_O_2_/H_2_O) = 1.76 V at pH = 7) [54] and/or hydroxyl radicals (through hydrogen peroxide) [39,55] can be expected because the superoxide radicals themselves are not able to take part in oxidation reactions. Both conduction and valence band potentials of the CN-M and CN-MCA materials were similar, which is why the reaction process was supposed to also be similar.

The direct reactions of holes with the antibiotics were also considered. The oxidation potentials of both compounds taken from literature [56,57] were recalculated to be against NHE at pH = 7: the values of 1.10 V for ofloxacin and 1.68 V for ampicillin were obtained. Comparing them with the conduction band potentials of CN-M and CN-MCA (Table 4), it implies that, unlike ampicillin, the holes can also take part in the photocatalytic decomposition of ofloxacin. This agrees with the photocatalytic experiments showing the larger decomposition of ofloxacin (Figure 5). The photocatalytic activity of CN-MCA was higher in both photocatalytic reactions, likely due to its larger SSA. Some possible interactions of the CN surfaces and the antibiotics were investigated by the measurement of electrokinetic potentials.

### 3.8. Measurement of Zeta Potentials

**Figure 6:**
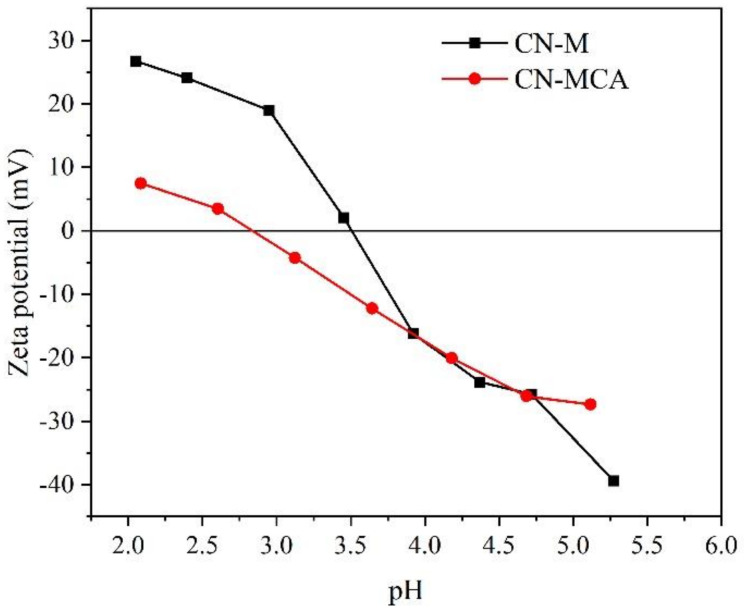
Zeta potentials of CN materials depending on pH.

The electrokinetic (zeta) potentials of the CN materials were measured depending on pH by titration of their aqueous suspensions with hydrochloric acid (see Figure 6). The plots indicate the presence of positive and negative species on the CN surfaces. The H^+^ ions can react with –NH_2_, >NH, =N- and –OH groups forming –NH_3_^+^, >NH_2_^+^, =NH^+^- and –OH_2_^+^ ones and with OH^-^ ions forming -NH^−^, =N^−^ and –O^−^ species, respectively

The plots in Figure 6 show that the CN zeta potentials were positive in the acid aqueous suspensions. The aqueous suspensions of CN-M and CN-MCA were titrated with HCl (0.025 mol L^−1^ and 0.25 mol L^−1^). The isoelectric points were found at 3.50 and 2.83 for CN-M and CN-MCA, respectively. One can see that the values corresponding to CN-M and CN-MCA were different, which is consistent with their different properties, as above-mentioned. The lower positive potentials of CN-MCA indicate the lower content of protonated amino and hydroxyl groups. During the photocatalytic experiments, pH decreased from 5 to 4 and thus the zeta potentials of both materials were similar. This implies that there were no different specific interactions of the CNs in their reactions. However, aampicillin carboxylic groups (pK_a1_ = 2.6, pK_a2_ = 7.1) [58] were more dissociated than ofloxacin ones (pK_a1_ = 6.2, pK_a2_ = 8.2) [58], which could lead to their higher repulsion from the negatively charged CN surfaces and its photocatalytic decomposition was less effective.

## 4. Conclusions

Two kinds of graphitic carbon nitrides were prepared by heating melamine and a melamine-cyanurate complex precipitated by mixing the solutions of melamine and cyanuric acid. Even when the thermal treatment of both precursors was carried out under the same conditions, the obtained materials were different in some of their properties. The specific surface area of CN-MCA (225 m^2^g^−1^) was about 19 times larger than that of CN-M (12 m^2^g^−1^) and the content of oxygen in CN-MCA was lower (0.62 wt.%) than in CN-M (1.88 wt.%). The band gap energy of CN-MCA was 2.73 eV and that of CN-M was 2.64 eV, likely to be a result of the CN-MCA structure exfoliation into nanosheets.

The photocatalytic activity of the CN materials was tested using the decomposition of antibiotics ofloxacin and ampicillin under the irradiation of 420 nm. The activity of CN-MCA was higher due to its larger SSA. The ofloxacin reaction obeyed the first-order kinetics in contrast to ampicillin, which was decomposed according to the zero-order one. The photocatalytic decomposition of ofloxacin was higher in the presence of both materials because superoxide radicals, together with the photoinduced holes, were able to take part in the reactions. In the case of ampicillin, the holes were supposed to be inactive. In addition, ampicillin, with more dissociated acidic groups, could be more repulsed from the CN surface.

Further studies will be focused on improving the CN-MCA material photocatalytic efficiency by using solar irradiation for different pharmaceutical degradation.

## Figures and Tables

**Table 1:** Elemental analysis of melamine-cyanurate complex (CN-MCA) materials.

Material	C (wt.%)	H (wt.%)	N (wt.%)	C/N	O (wt.%)
MCA	27.90	3.83	49.40	0.565	18.87
CN-M	34.93	1.72	61.47	0.568	1.88
CN-MCA	34.90	2.38	62.10	0.562	0.62

**Table 2:** Band gap energy and specific surface area of CN materials.

Material	*E_g_* (eV)	SSA (m^2^ g^−1^)
CN-M	2.64	12
CN-MCA	2.73	225

**Table 3:** Some XRD parameters of CN materials.

Material	2 Theta (deg)	FWHM(002) (deg)	L(002) (nm)	d(002) (nm)
CN-M	31.94	1.29	7.15	0.325
CN-MCA	32.00	1.44	6.41	0.325

Note: The 2 Theta values correspond to the (002) diffractions.

**Table 4:** Observed kinetic constants, E_CD_ and E_VB_ values of CN materials.

Material	k_obs_ (Ofloxacin) × 10^−3^ (min^−1^)	k_obs_ (Ampicillin) × 10^−3^ (mol L^−1^ min^−1^)	E_CD_ (eV)	E_VB_ (eV)
CN-M	17.9 ± 0.8	1.36 ± 0.05	−1.23	1.41
CN-MCA	41.7 ± 1.3	6.63 ± 0.21	−1.17	1.56

## Data Availability

Not applicable.

## Supplementary Materials

The following are available online at https://www.mdpi.com/article/10.3390/ma14081967/s1, Figure S1. Molecular structure of Ofloxacin; Figure S2. Molecular structure of Ampicillin.
