# Supplementary material for: Comparison of Graphitic Carbon Nitrides Synthetized from Melamine and Melamine-Cyanurate Complex: Characterization and Photocatalytic Decomposition of Ofloxacin and Ampicillin
Source: Materials (Basel). 2021 Apr 14;14(8):1967. doi: 10.3390/ma14081967 (PMC8070965; doi:10.3390/ma14081967)
Supplement: Supplementary file 1 [file materials-14-01967-s001.pdf]

Supplementary materials

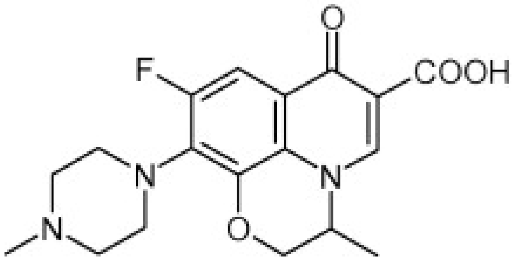

Figure S1. Molecular structure of Ofloxacin.

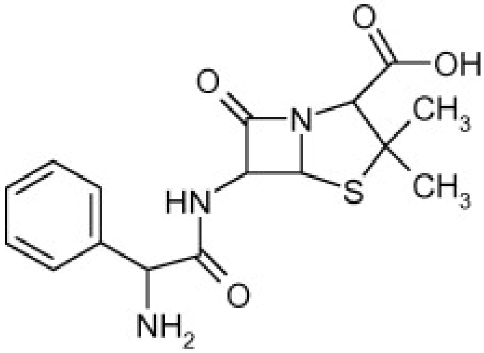

Figure S2. Molecular structure of Ampicillin.
